# *The market for amino acids*: understanding supply and demand of substrate for more efficient milk protein synthesis

**DOI:** 10.1186/s40104-020-00514-6

**Published:** 2020-11-12

**Authors:** Virginia L. Pszczolkowski, Sebastian I. Arriola Apelo

**Affiliations:** 1 Department of Animal and Dairy Sciences, University of Wisconsin-Madison, Madison, WI USA; 2 Endocrinology and Reproductive Physiology Graduate Training Program, University of Wisconsin-Madison, Madison, WI USA

**Keywords:** Amino acids, Blood flow, Insulin, Mammary uptake, Milk proteins, mTORC1, Nitrogen efficiency, Splanchnic tissues

## Abstract

For dairy production systems, nitrogen is an expensive nutrient and potentially harmful waste product. With three quarters of fed nitrogen ending up in the manure, significant research efforts have focused on understanding and mitigating lactating dairy cows’ nitrogen losses. Recent changes proposed to the Nutrient Requirement System for Dairy Cattle in the US include variable efficiencies of absorbed essential AA for milk protein production. This first separation from a purely substrate-based system, standing on the old limiting AA theory, recognizes the ability of the cow to alter the metabolism of AA. In this review we summarize a compelling amount of evidence suggesting that AA requirements for milk protein synthesis are based on a demand-driven system. Milk protein synthesis is governed at mammary level by a set of transduction pathways, including the mechanistic target of rapamycin complex 1 (mTORC1), the integrated stress response (ISR), and the unfolded protein response (UPR). In tight coordination, these pathways not only control the rate of milk protein synthesis, setting the demand for AA, but also manipulate cellular AA transport and even blood flow to the mammary glands, securing the supply of those needed nutrients. These transduction pathways, specifically mTORC1, sense specific AA, as well as other physiological signals, including insulin, the canonical indicator of energy status. Insulin plays a key role on mTORC1 signaling, controlling its activation, once AA have determined mTORC1 localization to the lysosomal membrane. Based on this molecular model, AA and insulin signals need to be tightly coordinated to maximize milk protein synthesis rate. The evidence in lactating dairy cows supports this model, in which insulin and glucogenic energy potentiate the effect of AA on milk protein synthesis. Incorporating the effect of specific signaling AA and the differential role of energy sources on utilization of absorbed AA for milk protein synthesis seems like the evident following step in nutrient requirement systems to further improve N efficiency in lactating dairy cow rations.

## Introduction

Since some humans evolved to digest lactose into adulthood around 7000 years ago [1], the nutrition of a continually expanding population has relied heavily on the ability of ruminant animals to convert fibrous vegetation into milk. An amazing symbiosis with rumen microbes allows ruminants to utilize structural carbohydrates and non-protein nitrogen (N) sources to an extent not possible for monogastric species. Rumen microbes can synthesize their own amino acids (AA) from non-protein N sources that the ruminant then absorbs to satisfy its own AA requirements. However, the capacity of rumen microbes limits the amount of microbial protein that the cow can obtain from ruminal fermentation [2]. In addition, microbial protein contains an essential AA profile [3] that, compared with milk proteins [4], generates deficiencies for some essential AA, including His, Leu, Trp, Val, and possibly Met. Therefore, to meet absorbed AA requirements for milk production, nutritionists supplement lactating dairy cow rations with rumen undegradable-protein (RUP) and rumen-protected AA.

Despite the impressive ability of the cow to convert low quality dry matter into milk, dietary N is converted to milk proteins with a disappointing efficiency of 25% to 27% on average [5], with the remaining three quarters of dietary N being lost in manure. The three largest losses of dietary N happen: 1) in the rumen, from ammonia not incorporated into microbial protein and subsequently converted to urea in the liver; 2) in the small intestine, from undigested microbial protein, RUP, and endogenous protein excreted in feces; and, 3) post-absorption, from AA absorbed in the small intestine but not used for production purposes [6]. Dijkstra and collaborators predicted a maximum microbial N efficiency of 90% with 25% of the N as nucleic acids and 85% microbial protein digestibility [6]. Based on those estimations, about 43% of the rumen degradable protein (RDP) is lost before being absorbed as AA. Post-absorptive losses are the most difficult to estimate because there is not a true measurement of the supply (i.e. absorbed AA), and because data on the partitioning of the losses between maintenance requirements and productive inefficiencies are both difficult to obtain and very limited. In fact, main nutrient requirement systems for lactating dairy cows present significant discrepancies in estimates of digestible AA supply, maintenance costs, and AA efficiency for milk protein production [6]. Regardless of how the losses are partitioned, more than 50% of the N absorbed as AA is not used for productive purposes (i.e. milk proteins). Therefore, post-absorption is where the largest of the three main N losses happen, and where we have the least understanding of how to mitigate them.

Nutrient requirement systems for several livestock species including lactating dairy cows predict production responses based on the genetic potential of the animal and the substrate (e.g. AA, glucose, etc.) supplied by the diet. In lactating dairy cows, milk protein yield is predicted based on an empirical factorial approach driven by the energy supplied as net energy for lactation, the pool of digestible AA (referred to as metabolizable protein (MP) in North American systems), and the relative content of the most limiting AA in this digestible AA pool [7]. This approach implies that substrate supply drives the synthesis of milk proteins, and when one of the factors is depleted (e.g. MP), milk protein synthesis stops. As a result, AA requirements are over-predicted throughout a wide range of supply, cows are overfed with protein, and a large amount of N is lost in manure [8]. This substrate-driven approach stands atop Mitchell’s and Block’s work on most limiting AA in rats [9], which led to the development of the long-standing limiting AA theory. Based on this theory, each biological function (e.g. milk production) requires an ideal AA profile, and only supplementation with the most limiting AA relative to that ideal profile can boost production. The limiting AA theory further stands on the law of the minimum, which states a similar concept but in regard to minerals in plant nutrition; and on Lavoisier’s law of conservation of mass. Correctly, the amount of amino-N or individual essential AA required to synthesize a given amount of milk protein must be supplied (or elsewise obtained from catabolism of body proteins) as substrate for translation, or those proteins cannot be synthesized. However, while the AA in the milk proteins do need to be supplied, the AA not used for production purposes, which represent more than 50% of absorbed AA supply [10], could be partially saved.

Modern nutrient requirement systems have started to shift towards using diminishing efficiencies [11–13], meaning that the efficiency of utilization of AA for milk protein synthesis decreases in relationship with AA supply, either as absolute amount or as a ratio with energy supply. Lapierre and collaborators have proposed major changes to the NRC system [7], including variable efficiencies for individual essential AA [14]. This is a significant shift in the approach to predicting milk protein yield and reducing N losses because it recognizes the ability of the cow to adjust its own AA efficiency. We hypothesize that further improvements in N efficiency would come by switching from a substrate-driven system in which milk protein synthesis rate is determined by the most limiting nutrient, to a demand-driven system in which the rate of milk protein synthesis is determined primarily by the mammary glands. We will show that mammary cells integrate an array of physiological signals, including certain essential AA, energy, and hormones, aiming to demonstrate the large control they have not only on milk protein production, but also on removal of AA from blood. Given the critical role of splanchnic tissues on AA metabolism, we will also analyze how strategies to maximize mammary AA demand affect utilization of AA by splanchnic tissues.

## Signaling regulation of milk protein synthesis

### The mTORC1 pathway

Nutritional control of milk protein output falls on the regulation of mRNA translation. One cellular pathway that has been established as integral for the regulation of milk protein translation is centered around the mechanistic target of rapamycin complex 1 (mTORC1). This kinase complex is composed of the serine/threonine protein kinase mTOR and its binding partners: mammalian lethal with SEC13 protein 8 (mLST8), required for complex assembly; regulatory associated protein of mTOR (Raptor), a substrate-binding protein; Dishevelled, Egl-10 and Pleckstrin domain domain-containing mTOR interacting protein (DEPTOR), a regulatory protein; and 40-kDa proline-rich Akt substrate (PRAS40) [15]. Active mTORC1 phosphorylates downstream substrates including, among others, ribosomal protein S6 kinase β-1rp (S6K1) [16], eukaryotic translation initiation factor 4E-binding protein 1 (4E-BP1) [17], and Unc-51-like autophagy activating kinase (ULK1) [18]. By controlling activity of its substrates, mTORC1 stimulates anabolic processes including ribosomal biogenesis and mRNA translation and represses catabolic ones like autophagy. Its anabolic role in cellular metabolism explains why nutritional factors are key activators of mTORC1.

Amino acids stimulate the Ragulator complex to recruit mTORC1 to the lysosomal membrane [19], where the complex must be located to subsequently be activated by the small GTPase Rheb [20]. The mechanisms by which individual AA signal for Ragulator activation and mTORC1 recruitment to the lysosome have begun to be elucidated, including sensing of Leu by Sestrin2 [21], of Arg by arginine sensor for mTORC1 subunit 1 (CASTOR) [22], and of the Met metabolite *S*-adenosylmethionine (SAM), by the SAM sensor upstream of mTORC1 (SAMTOR) [23]. Other essential AA at physiological or sometimes supraphysiological concentrations have been reported to stimulate mTORC1, but sensing mechanism for those essential AA have not been elucidated. The Leu sensor Sestrin2 has been identified as an integral regulator of casein synthesis, controlling more than 50% of the response to AA [24]. Similarly, a mechanistic quantitative model of casein synthesis fully controlled by mTORC1 explained almost 50% of the variation observed in casein synthesis [25]. A recent proteomic analysis of the lysosomal membrane revealed that only 58% of membrane binding proteins overlap between bovine mammary epithelial cells and rat hepatocytes [26, 27]. Therefore, although key components are generally conserved, caution is still required when extrapolating mTORC1 functions between species and tissues, or from *in vitro* to *in vivo* systems.

This model of activation of mTORC1 indicates that AA only play a role in recruiting mTORC1 to the lysosomal membrane, where mTORC1 is then activated by Rheb [20]. Rheb localization to the lysosome is blocked by an active, lysosome-docked tuberous sclerosis protein complex (TSC) [28]. This inhibition of Rheb by TSC is reversed by phosphorylation of the TSC component TSC2, by Akt in response to insulin and growth factors [29]. Furthermore, TSC is positively regulated by 5′ adenosine monophosphate-activated protein kinase (AMPK), which senses low cellular energy via AMP [30]. However, in bovine mammary epithelial cells AMPK seems to have limited effect on mTORC1 activity and casein synthesis [31]. This mechanism integrates insulin and AA signals to stimulate anabolic functions, like milk protein synthesis, and therefore cellular nutrient demand. However, this model has been challenged in mammary epithelial cells, where essential AA have shown independent effects on mTORC1 signaling and casein synthesis, rather than a synergistic interaction with insulin [32]. On the other hand, *in vivo*, insulin or abomasal infusion of starch has been shown to potentiate the effect of AA on milk protein production [33, 34].

Activity of the mTORC1 pathway is correlated with milk proteins expression *in vitro* [35] and *in vivo* in lactating mice [36], and milk protein yield in lactating dairy cows [34]. However, as yet a causal link between mammary mTORC1 activity and casein synthesis *in vivo* has not been demonstrated. Some studies in cows even posit contradictory activation of mTORC1 activity and milk protein production, with diminished Lys supply increasing S6K1 phosphorylation even as protein yield is decreased [37]. Data collected from lactating mice in our laboratory point toward mTORC1 as a major regulator of lactation. Using a murine model of lactation, we found that dietary AA were unable to sustain lactation performance when dams were treated with the mTORC1 inhibitor rapamycin [38]. Rapamycin treatment of dams under adequate protein diets decreased pup growth to the level seen with dams fed a 50% protein-restricted diet, with average pup weight 15% below control by the end of the lactation and corresponding to reductions in mammary mTORC1 signaling.

Overall, mTORC1 is clearly involved in milk protein synthesis. Certain AA, notably Met and Leu, are responsible for mTORC1 docking at the lysosomal membrane, where it can then orchestrate a cellular anabolic response. The evidence in non-mammary cell types, supported by *in vivo* responses in lactating dairy cows, indicates that without insulin signaling, AA alone are unable to fully activate mTORC1. Therefore, evolutionarily selected nutritional signals are essential for determining the mammary demand of AA for milk protein synthesis through mTORC1.

### The Integrated Stress Response

Unlike mTORC1 that responds to nutritional signals promoting mRNA translation, the integrated stress response (ISR) pathway senses cellular stressors and represses translation of new proteins. The ISR revolves around the eukaryotic translation initiation factor 2 (eIF2), a G-protein and mRNA translation initiation factor responsible for delivering the first, Met-loaded tRNA to the small ribosomal subunit to initiate a new cycle of translation initiation [39]. The Α subunit of eIF2 can be phosphorylated on the same residue (Ser51) by four kinases that each sense different types of stress [40]: protein kinase double-stranded RNA-dependent (PKR), activated by viral RNA; heme-regulated inhibitor (HRI); protein kinase R-like ER kinase (PERK), induced by misfolded proteins in the endoplasmic reticulum; and general control non-derepressible-2 (GCN2), activated by unloaded tRNAs. Phosphorylated eIF2α then represses eIF2B, its own GTP exchange factor, preventing GDP release and in turn, GTP binding to eIF2. Therefore, by repressing eIF2 GTPase activity, these four kinases can control the rate of global mRNA translation.

The two eIF2α kinases most clearly related to AA metabolism are GCN2 and PERK, the latter as part of the unfolded protein response (UPR), discussed below. Meanwhile, GCN2 senses AA deficiencies through binding to uncharged tRNA, previously reviewed [8]. There is a lack of conclusive evidence on the role of eIF2α and its kinases GCN2 and PERK on milk protein synthesis regulation. In lactating mice, protein or individual AA restriction did not affect eIF2α mRNA and protein expression or protein phosphorylation, even though these diets did affect litter growth rates [36]. In bovine mammary tissue slices, individual AA had mild non-significant effects on eIF2α phosphorylation, and only the combination of some or all the essential AA significantly altered eIF2α phosphorylation [41, 42]. Similarly, in lactating dairy cows, only total essential AA, by subtraction or supplementation, significantly altered eIF2α phosphorylation, but again with no effects by individual AA [43].

In the studies described above, the effect of essential AA on eIF2α phosphorylation would logically be mediated by GCN2, as AA availability was altered. Interestingly, essential AA supplementation also increased the expression of eIF2Bε, one of the subunits of the exchange factor that activates eIF2 [37, 43], potentially contributing to translation activity. Phosphorylation of this subunit can increase or decrease its exchange factor activity, depending on the kinase and residue, also altering the rate of mRNA translation [44, 45], but in the studies reported above using lactating dairy cows, phosphorylation was not significantly affected [37, 43].

### The Unfolded Protein Response

As the site of translation for milk proteins and of the synthesis of milk fat globules, the endoplasmic reticulum (ER) is an important structure for milk synthesis. Generally, when the ER experiences stress resulting in an overload of unfolded proteins, a proteostatic system termed the unfolded protein response (UPR) is deployed to either return the cell to homeostasis or induce apoptosis, depending on whether or not the ER stress can be overcome [46]. The UPR has three branches that work to regain proteostasis by decreasing the protein folding load on the ER and increasing its capacity. The first of these, PERK, phosphorylates eIF2α which subsequently acts to downregulate global protein synthesis while simultaneously upregulating translation of activating transcription factor 4 (ATF4) [47]. We discuss eIF2α and ATF4 in further detail in a subsequent section. As mentioned in the previous section, PERK is also one part of the ISR. The second branch, activating transcription factor 6 (ATF6), induces expression of UPR-specific mRNAs, including ER chaperones and the transcription factor X-box protein 1 (XBP1) [48]. Finally, the kinase and endoribonuclease inositol-requiring enzyme 1α (IRE1α) can both splice XBP1 mRNA to increase ER protein folding capacity [49]. Beyond responding to ER stress, the UPR has been established as responsive to non-stress-induced stimuli for broader regulation of cellular homeostasis.

In the lactating mammary gland, the UPR has a variety of functions, not all of which are yet understood. Similar to other secretory cell types, in mammary epithelial cells the UPR is required for terminal differentiation into the secretory phenotype [50]. As with mTORC1 and the ISR, the UPR’s role in mammary AA metabolism is currently under investigation. As yet, there is no clear mechanism by which AA might regulate the UPR itself, save for other pathways promoting increased mRNA translation and ER load. Increased splicing of XBP1 mRNA concomitant with an increase in milk protein synthesis has been observed in the mammary glands of lactating cows in response to 5-day abomasal infusion of essential AA yield [51]. Interestingly, lipogenic but not glucogenic energy seems to blunt the effect of AA on XBP1 mRNA splicing [52]. In rat liver, IRE1α and XBP1 mRNA splicing is activated post-prandially in an mTORC1-dependent manner [53]. Although mTORC1 signaling was not affected in Nichols’ study [51], this hepatic mTORC1-dependency observed in rats may potentially offer a partial explanation for how XBP1 splicing was altered in these cows in response to essential AA. Bidirectional-crosstalk between the mTORC1 and UPR systems likely is involved in orchestrating how the mammary gland metabolizes AA. Similarly, removal of milk from the glands is a known regulator of production, probably through a UPR dependent mechanism. Increased milking frequency increased milk and milk components production independently of gland distension [54, 55]. At least one constituent of milk, a whey protein termed the feedback inhibitor of lactation (FIL), has been described as the cause for this response both *in vitro* and in several lactating species. Administration of FIL to mammary epithelial cells results in changes to the endoplasmic reticulum morphologically consistent with ER stress and the UPR [56, 57].

The interconnectedness between mTORC1, ISR, and UPR pathways highlights the complexity of milk protein synthesis regulation. Although mTORC1 is an anabolic cellular hub, while ISR and UPR are more associated with catabolic responses, environmental insults and stimuli induce a coordinated response of the three pathways to maintain proteostasis. However, if unlike mTORC1, the ISR and UPR in the mammary glands can only respond to dietary protein or total essential AA rather than individual ones, their exploitation as mechanisms to increase N efficiency in lactating dairy cows may be limited.

Until now, we briefly described mechanisms that sense environmental and physiological signals that control the rate of milk proteins translation, which set the demand for AA. A successful demand-driven system must not only set the rate of protein synthesis, it also needs to secure the AA supply for that set demand. Mammary extraction of AA from blood is determined at tissue level by: 1) the supply of AA in blood, further determined by the concentration of AA in blood and the flow rate to the glands; 2) the number of secretory cells that extract those AA and produce milk proteins; 3) and by the ability of existing secretory cells to remove those AA from blood and secure them into milk proteins.

## Mammary blood flow

Blood AA concentrations are determined by the rate of appearance of individual AA into the circulation, which is in turn the result of AA absorption from the lumen of the intestine minus retention by the splanchnic tissues. However, arterial blood supplies about 80% of the AA removed by splanchnic tissues [58], such that the peripheral tissues’ extraction of AA largely determines the supply to the splanchnic tissues. Independent of the amount of AA absorbed, changes in local blood flow control partitioning of those AA between peripheral tissues, and can affect the supply of AA to the mammary glands [59]. In high producing dairy cows, the mammary glands can remove more than three quarters of the AA released by the splanchnic tissues [60], giving the mammary glands a deciding role in their own AA supply.

### Endocrine regulation of mammary blood flow

In the short term, mammary blood flow is positively regulated by nitric oxide through a feedback signal from adenosine, which indicates a decrease in cellular energy, and is negatively regulated by glucose [61]. On the other hand, longer-term glucose or starch infusion increased mammary blood flow [34, 62, 63]. Insulin, increased in plasma in response to glucose and starch infusion [34, 62], has been reported to stimulate nitric oxide synthase (eNOS) activity through phosphorylation by Akt [64]. Although insulin does not show an effect on blood flow in the short-term [61], a four-day hyperinsulinemic-euglycemic clamp in lactating dairy cows increased mammary blood flow [33], supporting the idea that nitric oxide synthase activity is also regulated by insulin in the mammary glands.

Nitric oxide production in mammary epithelial cells is also regulated by prolactin through a calcium mediated mechanism [65]. In addition, the neurotransmitter serotonin (5-hydroxytriptamine) regulates nitric oxide production via signaling through serotonin receptors [66]. Serotonin has been shown to regulate mammary blood flow in lactating animals [67]. During lactation, most of the serotonin is produced by the mammary glands from Trp through a prolactin-dependent mechanism [68]. In mice, prolactin also stimulates β-cell proliferation and insulin secretion through serotonin signaling [69]. In one study in late-lactation dairy cows, infusion of the serotonin precursor 5-hydroxytryptophan had the opposite effect, decreasing concentration of plasma insulin [70]. Controlling serotonin production, insulin secretion, and blood flow by prolactin may integrate nutrient partitioning towards the mammary glands in lactating animals.

### Amino acid regulation of mammary blood flow

The molecular mechanisms and overall effect of AA in mammary blood flow are far less clear than for insulin. In the short-term, AA infusion linearly increased mammary blood flow [61]. However, longer-term infusions of casein or essential AA have not shown an effect on mammary blood flow [34, 60, 62, 71]. Specific AA have not shown conclusive effects either. A combined infusion of the three most limiting AA — Lys, Met and His — into the jugular vein for 8 days had no effect on blood flow despite increasing milk protein yield [72]. Similarly, incremental doses of rumen-protected Met alone had no effect in mammary blood flow [73]. On the other hand, removal of the three most limiting AA individually from a complete AA infusate in lactating goats increased mammary blood flow by 33% for His [74], 40% for Met [75], and 41% for Lys, the latter with a concomitant three-fold increase in nitric oxide concentration in the mammary vein [76]. A plausible mechanism for the increase in blood flow in response to Lys restriction would be an increase on Arg uptake, the substrate for nitric oxide and co-substrate with Lys of the cationic AA transport systems. However, in that study, Arg uptake numerically decreased in response to Lys restriction [76]. In a separate study in lactating cows, Arg restriction decreased blood flow but Arg supplementation tended to have a negative effect on blood flow as well [77], which does not suggest a substrate effect of Arg for nitric oxide synthesis but more so an effect of AA imbalance. Remarkably, despite the indicated increases in mammary blood flow, restriction of His, Lys, or Met reduced milk protein yield. Therefore, without understanding how arterial deficiency of individual AA, or specifically Lys, affect mammary blood vessel nitric oxide concentration and blood flow, we will not be able to utilize those mechanisms to increase AA delivery to the mammary glands and N efficiency without compromising milk protein yield.

Conversely, supplementation with the mTORC1 signaling AA Leu and Ile increased mammary blood flow, which was partially responsible for an increase in total AA uptake and milk protein yield [72]. Therefore, although the mechanisms of mammary blood flow regulation are not yet clear, stimulatory signals of the mTORC1 pathway like insulin and branched chain AA seem to be promising strategies to increase nutrient partitioning towards the mammary glands.

## Mammary amino acid uptake

Uptake of AA by the mammary glands can change for either of two reasons: because the supply in blood changes, or because the mammary glands actively alter their affinity for a given AA, either increasing the glands capacity or altering enzyme activity. A classic example of a change in mammary affinity for individual AA is observed in the study by Bequette and collaborators in goats, referenced above. In addition to increasing mammary blood flow, restricting His resulted in a 43-fold increase in mammary affinity for that AA, concomitant with a decrease in the affinity for other AA [74]. A purely substrate-driven system cannot explain the observed production response, otherwise milk protein yield would have dropped proportionally to the 90% drop in plasma His observed. Instead, the mammary cells drastically increased their affinity for His to meet the demand for milk protein synthesis, which only dropped by 18%. More recently, a similar response was demonstrated with Met restriction, in which a 15-fold increase in mammary clearance of Met corresponded to decreases in the clearance of other AA [75]. This basic research gives clear evidence that the mammary glands can indeed change their affinity for individual AA. However, it does not provide a nutritional strategy to increase mammary nutrient uptake while maintaining or increasing milk protein yield.

A more feasible strategy to increase digestive AA efficiency without compromising the yield of milk proteins would be to supplement rather than remove nutritional factors that increase mammary affinity and extraction efficiency of AA. An example of that approach is the study by Yoder and collaborators [72]. In that case, jugular infusion of Leu and Ile not only increased mammary uptake of several AA due to an increase in blood supply, but also increased mammary affinity for and uptake of His, one of the limiting AA in MP, and Arg [72]. In the same experiment, a combined infusion of His, Lys, and Met also increased milk protein yield. Fascinatingly, this happened without increasing net uptake of two of the infused AA, His and Met, for which mammary affinity dropped by 30% and 70% respectively. Meanwhile, the affinity for Lys, the most limiting AA in that diet, was maintained, increasing the uptake of the AA to meet the output in milk. The infusion of those three AA also reduced the uptake-to-output ratio of other AA, possibly to support the increase in milk production [72]. While the latter response could be explained by the limiting AA theory, the response to Ile and Leu cannot. Instead, adaptive physiologic responses to treatment resulted in greater blood flow, and in turn supply and uptake of total AA, and mammary affinity for non-infused AA. Fascinatingly, when both substrate and stimulatory AA were provided, the response in milk protein yield was 46% greater than a strictly additive effect [72].

### Signaling regulation of mammary amino acid uptake

The plasticity of the mammary glands to alter their own blood flow and affinity for AA suggests that the mechanisms sensing AA supplies not only set the rate of protein synthesis but also adequately alter AA uptake to meet that demand. While the evidence is strong in regard to mTORC1 regulation of milk protein synthesis, it is less clear how an established demand of AA for milk protein synthesis induces higher rates of AA uptake by the mammary glands. A nonlinear relationship with arterial AA concentrations suggest that AA removal is not simply based on concentration gradients between the extra- and intracellular spaces [78]. In line with this nonlinear relationship, pharmacological repression of mTORC1 activity in a human embryonic kidney cell line (HEK293T) showed that mTORC1 controls gene expression of several AA transport systems expressed in mammary tissue, including the branched chain AA transporter L-type amino acid transporter 1 (LAT1) and cationic AA (Arg and Lys) transporter 1 (CAT1) [79]. In addition to gene expression, mTORC1 controlled membrane localization of LAT1 and sodium coupled neutral amino acid transporter (SNAT2) in human trophoblast cells [80].

Methionine, one of the most limiting AA in modern dairy cow diets, stimulates the expression of several AA transporter systems, also regulated by mTORC1. In bovine mammary epithelial cells, Met increased protein expression of LAT1 through an mTORC1-dependent mechanism [81–83]. Importantly, LAT1 further transports intracellular Leu into the lysosome where it can stimulate mTORC1 through v-ATPase [84]. Methionine also controlled the expression of two neutral AA transporters in bovine mammary epithelial cells: SNAT2, which transports small neutral AA such as Ala, Ser, and Gln; and alanine serine cysteine transporter 2 (ASCT2), which transports Ala, Ser, Cys, Thr, and Gln, both by mTORC1-mediated mechanisms [85, 86]. Regrettably, while plasma concentration of Met averages 21 μmol/L [87], *in vitro* effects of Met on mammary epithelial cell expression of AA transporters have only been studied at supraphysiological concentrations (300–600 μmol/L), leaving uncertainty as to the effect of Met *in vivo*. In fact, the evidence *in vivo* does not support the idea that Met alone can stimulate uptake of other AA by the mammary glands. In one study, infusion of Met did not affect extraction of AA by the mammary glands of early lactation dairy cows, while it decreased mammary uptake for most essential AA in mid lactation [88]. Meanwhile, in lactating goats, Met restriction tended to decrease uptake of most essential AA [75].

Unlike Met, jugular infusion of Leu, a stronger activator of mTORC1, increased mRNA and protein expression of the cationic AA transporter CAT1 in porcine jejunal epithelial cells [89], which is also expressed in the mammary glands of lactating cows [90], and shown to be regulated by mTORC1 [79]. In porcine jejunal cells as well, supraphysiological concentrations of Leu stimulated mRNA and protein expression of the small AA transporter ASCT2 [91], and in rat muscle, Leu stimulated SNAT2 mRNA expression, both also regulated by Met. Also in rat muscle, Leu stimulated mRNA expression of system A transporters in an mTORC1-dependent fashion [92, 93]. Although these are a crucial AA transport system for essential and non-essential AA, there is little or no evidence for mTORC1-dependent AA regulation of other AA transporters. Beyond expression levels, transporters localization and activity in the mammary glands has not been extensively study either.

The translation repressor eIF2α also stimulates the expression of several AA transport systems, including LAT1, CAT1, SNAT2, ASCT2, and anionic system xCT [94–98], possibly as a mechanism to overcome an AA deficiency detected by one of its regulatory kinases, GCN2. In addition to repressing overall mRNA translation, eIF2α stimulates translation ATF4, which translocates to the nucleus and binds to AA response elements, controlling the expression of the indicated transporters [47]. However, the inconclusive response of eIF2α to AA in the mammary glands leaves uncertainty of the role that ATF4 plays in the observed changes of mammary affinity for individual AA. Interestingly, ATF4 is also regulated by mTORC1 in an eIF2α independent fashion in HEK293T cells [79], possibly explaining the broad control of mTORC1 on AA transporters expression.

### Endocrine regulation of mammary amino acid uptake

Insulin has a remarkable number and variety of functions in lactating animals beyond its canonical role in regulating glucose homeostasis, from initial lactogenesis [99] to promotion of milk protein synthesis [100]. Insulin also affects mammary epithelial cell affinity for individual AA, mainly group 2 essential AA. These are AA metabolized by the mammary glands, and as a result, group 2 AA have an uptake to output in milk ratio greater than one [101]. A hyperinsulinemic-euglycemic clamp not only directed more nutrients towards the mammary glands by increasing mammary blood flow, it also increased extraction efficiency of group 2 AA and milk protein yield [33]. Similarly, two-week abomasal infusion of 1.5 kg of glucose increased mammary extraction and metabolic efficiency of group 2 essential AA and milk protein yield [62]. In the same study, infusion of 695 g of AA with the profile of casein also increased milk protein yield but through completely different mechanisms. Unlike glucose, infusion of AA did not affect mammary blood flow and reduced mammary extraction and metabolic efficiencies, resulting in significantly larger catabolism of AA and N losses [62]. Interestingly, infusion of glucose combined with AA increased the response in milk protein yield 40% greater than would be expected if the effects were additive. Meanwhile, infusion of 1 kg of glucose for 5 days failed to significantly increase mammary blood flow, among group 2 AA only increased affinity for Leu and Ile, and did not affect milk protein yield [71]. Separately, abomasal infusion of starch also increased mammary affinity for group 2 AA, plus Phe, Trp and several non-essential AA [34]. In line, dietary supplementation of starch in Jersey cows, as compared to an isoenergetic fibrous diet, also increased arterial insulin, mammary extraction, and metabolic efficiencies of group 2 AA, this time without an effect on mammary blood flow [102, 103].

The mechanisms of insulin and insulinemic energy regulation of mammary AA affinity remains unclear. Most important AA transporters on the observed effect of insulin would be system L that transports branched chain AA and CAT1 that transport the cationic AA Arg and Lys. In human trophoblasts, glucose and insulin stimulated system L activity in an mTORC1 dependent fashion [104]. Similarly, in bovine mammary explants, insulin increased system L gene *SLC7A5* that encodes for LAT1 protein, and increased Lys uptake [105]. In mammary tissue from lactating mice, insulin increased Arg transport by the Na^+^-independent anionic transporter CAT1 [106].

The lactogenic hormone prolactin has also been shown to stimulate amino acid transporter expression and activity. In bovine mammary epithelial cells, prolactin increased LAT1 expression [107]. In mammary tissue of lactating mice, prolactin increased Arg transport by CAT1 [106], and in lactating rats, it increased mammary expression of the neutral amino acid transporter SNAT2 [108]. Although plasma prolactin concentration can be modified by diet [109], it is still uncertain if increasing prolactin concentration can be part of a dietary strategy to efficiently increase mammary retention of AA and milk protein yield.

As the canonical indicator of systemic energy status, it is unsurprising that insulin has an important role in regulating the energy-intensive activity of producing milk. Insulin secretion and activity are unique in ruminating animals due to their digestive anatomy and physiology, but the molecular mechanisms for insulin signaling intracellularly are conserved across species. Insulin has been shown to increase mammary AA uptake and sequestration into milk proteins both by increasing blood flow (and therefore AA supply) to the mammary glands and by increasing mammary AA affinity. Aggregating knowledge from different species and cell types directs towards a coordinated effect of insulin on AA uptake and milk protein synthesis mediated by mTORC1, while the effect of insulin on blood flow could be mTORC1 independent.

## Splanchnic utilization of amino acids

Estimates indicate that splanchnic tissues sequester half of the essential AA absorbed from the small intestine [58]. However, in addition to AA absorbed from the intestine, splanchnic tissues receive AA in arterial blood, which represent about 80% of the total AA flux to the tissue bed [8]. At least in sheep, the portal drained viscera (PDV) does not seem to prioritize between AA coming from luminal absorption or the arterial blood, except for the case of Phe [110]. In all species, the liver cannot prioritize between luminal and arterial AA because both are mixed in the portal vein. Therefore, splanchnic tissues are the primary competitor for AA to the mammary glands.

As opposed to the unequivocal ability of the mammary glands to alter AA use in response to different nutrients and endocrine signals, particularly insulinemic energy, the response of the splanchnic tissues to similar insults is far less conclusive. Hanigan and collaborators previously proposed fractional (i.e. fixed) extraction efficiencies of AA by both the PDV and the liver [111, 112]. A recent revision of those models revealed that similar to the mammary glands, PDV utilization of individual AA are better represented with saturable functions, suggesting a fixed requirement for AA [58]. Conversely, liver extraction of Arg, His, Lys, Met and Phe were best represented by non-linear functions, with extraction efficiencies positively related to AA supply [58]. This means that the more of these AA the liver receives, the more it clears, in line with the liver’s role to dispose of excess absorbed nitrogen. Branched chain AA were not significantly cleared by the liver (i.e. < 1% extraction) and Thr had a negative adjustment parameter, indicating saturable extraction as for the PDV. Minimum prediction errors suggest that mechanisms other than supply play little or no role in the regulation of hepatic extraction of AA.

Cantalapiedra-Hijar and collaborators assessed how dietary energy from starch vs fiber impacts hepatic extraction of AA at 12% and 16.5% crude protein (CP) diets in lactating Jersey cows [102]. In line with the most recent fitting of Hanigan’s model [58], dietary CP but not energy source affected hepatic extraction of most AA. Conversely, dietary starch, probably through an insulin-mediated effect, increased essential AA removal by the mammary glands in these same cows [103], resulting in lower recycling and greater release of absorbed AA by splanchnic tissues. A more aggressive approach than dietary starch (i.e. abomasal infusion of 1.5 kg glucose/d) reduced hepatic fractional extraction and flux of AA, despite increased hepatic blood flow and coinciding with a milk protein yield increase of 75 g/d [62]. Likely, discrepancies between these two studies [62, 102] on the ability of the liver to alter its release of AA to the periphery have to do with energy level (isoenergetic vs increased) and energy source (dietary starch vs infused glucose). Clearly, improved glucogenic energy supply has a positive impact on the availability of AA to the mammary glands, but the molecular mechanisms in the liver and splanchnic tissues for regulating AA usage are not yet settled. In male goats fed diets containing 10% or 30% non-fiber carbohydrate, liver transcriptomic analysis revealed that the liver responded to increased energy supply by downregulating many of the genes responsible for ureagenesis, in correlation with upregulation of insulin signaling genes [113]. Similarly in lactating cows, a hyperinsulinemic-euglycemic clamp decreased plasma urea concentration [114].

Unlike the mammary glands and the PDV, the liver has been shown to extract AA proportionally to its supply, of which 80% is from recirculating arterial blood. Although less research exists on the splanchnic tissues than on the mammary gland in this regard, the evidence points toward insulin and insulinemic energy behaving oppositely on hepatic extraction of AA compared to the mammary glands. Most likely, this is not because of different signaling mechanisms between the two organs, but rather because of their metabolically opposite directives, with the liver acting catabolically (AA to urea) and the mammary glands anabolically (AA to protein). In this way, both tissues work in tandem under the same set of systemic signals to meet metabolic needs while maintaining systemic homeostasis.

## Conclusions

In this review we have discussed physiological and molecular mechanisms that pertain to metabolism of AA during lactation. The prime directive of the lactating mammary glands is to make milk, and to accomplish this goal the glands must be able to sense and respond to nutrient and fuel supply. Although all the genetically encoded AA are required for the synthesis of milk proteins, an evolutionarily selected group of essential AA plays a unique signaling role, communicating substrate availability. Beyond the AA themselves, other metabolic signals also control the synthetic process. Among them, here we present insulin not only as the canonical signal of systemic energy status, but also as key regulator of AA metabolism, controlling AA supply, mammary uptake, and utilization in protein synthesis. Transduction pathways like mTORC1, ISR, and UPR integrate this complex matrix of signals into a set rate of translation that, to yield given amount of protein, will generate a demand for AA. The evidence in mammary epithelial cells as well as other cell types suggest that the same transduction pathways that set the rate of protein synthesis also manipulate AA transport systems to guarantee the supply of substrate, maintaining cellular proteostasis.

Previous lack of evidence regarding this coordination at cellular level resulted in nutrient requirement systems purely based on substrate supply. Including variable efficiencies for individual AA, as proposed by Lapierre and collaborators [14], shifts the approach from substrate-defined to incorporating the known ability of the cow itself to alter AA efficiency. A blanket definition of “energy” that includes any source of calories regardless of its metabolic or endocrine signaling capacity is all that nutritionists currently have to work with. New evidence in lactating cows suggest that the type (i.e. glucogenic vs lipogenic), rather than the density, of energy sources dictates the production response and ability for cows to utilize AA. Insulin signaling, required for mTORC1 activity *in vitro*, appears to be important in explaining the differential responses to types of energy on AA use, both in non-bovine models and in lactating cows themselves. The splanchnic tissues, significant in part because of their anatomical position between nutrients incoming from the gastrointestinal tract, may be an underexplored system for manipulation of mammary AA availability and utilization. Hepatic functions like ureagenesis, the process by which the liver converts excess N into urea for disposal as urine and a significant route for N loss in dairy cattle, may offer opportunities for improving N efficiency and post-hepatic AA availability through dietary manipulation of insulin signaling.

We foresee the integration of substrate and signaling effects of nutrients and other environmental insults (e.g. thermal stress) as the major challenge for the next generation of nutrient requirement systems for lactating dairy cows. Hopefully, once this challenge is overcome, future lactating dairy cow diets will be formulated with less excess of the nutrients harvested in milk, and will be strategically supplemented with the signaling nutrients that maximize productive efficiency. In this way, both feed inputs and waste outputs can be minimized.

Generation of molecularly informed models like that for casein synthesis in mammary epithelial cells proposed by Castro and collaborators [25] is likely to be key for organizing and understanding the complex system of mammary AA metabolism. Integrating regulation of mammary and splanchnic blood flow and nutrient uptake to meet the demand generated intracellularly, will be critical to better predict production responses and to maximize nutrient efficiency for milk production.

## Data Availability

All data generated or analyzed during this study are included in this published article.

## Abbreviations

4E-BP1: 4E-binding protein 1
AA: Amino acids
AMP 5′: Adenosine monophosphate
AMPK 5′: Adenosine monophosphate-activated protein kinase
ASCT2: Alanine serine cysteine transporter 2
ATF4: Activating transcription factor 4
ATF6: Activating transcription factor 6
CASTOR: Cytosolic arginine sensor for mTORC1 subunit 1
CAT1: Cationic amino acid transporter 1
CP: Crude protein
DEPTOR: Dishevelled, Egl-10 and Pleckstrin domain domain-containing mTOR interacting protein
eIF2: Eukaryotic translation initiation factor 2
eIF2α: Eukaryotic translation initiation factor 2α
eIF2B: Eukaryotic translation initiation factor 2B
eNOS: Endothelial nitric oxide synthase
ER: Endoplasmic reticulum
GCN2: General control non-derepressible-2 ISR integrate stress response
HEK293T: Human embryonic kidney cell line 293 T
HRI: Heme-regulated inhibitor
IRE1α: Inositol-requiring enzyme 1α
JNK: c-Jun NH2-terminal kinase
LAT1: l-type amino acid transporter 1
mLST8: Mammalian lethal with SEC13 protein 8
MP: Metabolizable protein
mTOR: Mechanistic target of rapamycin
mTORC1: Mechanistic target of rapamycin complex 1
mTORC2: Mechanistic target of rapamycin complex 2
N: Nitrogen
PDV: Portal-drained viscera
PERK: Protein kinase R-like ER kinase
PRAS40: 40-kDa proline-rich Akt substrate
PKR: Protein kinase double-stranded RNA-dependent
Raptor: Regulatory associate protein of mTOR
RDP: Rumen-degradable protein
RUP: Rumen-undegradable protein
S6K1: S6 kinase beta-1rp
SAM: *S*-adenosylmethionine
SAMTOR: *S*-adenosylmethionine sensor upstream of mTORC1
SNAT2: Sodium-coupled neutral amino acid transporter 2
TSC: Tuberous sclerosis complex
TSC2: Tuberous sclerosis complex 2
ULK1: Unc-51-like autophagy activating kinase
UPR: Unfolded protein response
XBP1: X-box protein 1
